# Linking Emotional Reactivity Between Laboratory Tasks and Immersive Environments Using Behavior and Physiology

**DOI:** 10.3389/fnhum.2019.00054

**Published:** 2019-02-18

**Authors:** Heather Roy, Nick Wasylyshyn, Derek P. Spangler, Katherine R. Gamble, Debbie Patton, Justin R. Brooks, Javier O. Garcia, Jean M. Vettel

**Affiliations:** ^1^United States Army Research Laboratory, Aberdeen Proving Ground, Adelphi, MD, United States; ^2^Department of Bioengineering, University of Pennsylvania, Philadelphia, PA, United States; ^3^Department of Psychological and Brain Sciences, University of California, Santa Barbara, Santa Barbara, CA, United States

**Keywords:** emotion, emotional reactivity, stress, anxiety, immersive reality, heart rate variability, EEG, neural connectivity

## Abstract

An event or experience can induce different emotional responses between individuals, including strong variability based on task parameters or environmental context. Physiological correlates of emotional reactivity, as well as related constructs of stress and anxiety, have been found across many physiological metrics, including heart rate and brain activity. However, the interdependances and interactions across contexts and between physiological systems are not well understood. Here, we recruited military and law enforcement to complete two experimental sessions across two different days. In the laboratory session, participants viewed high-arousal negative images while brain activity electroencephalogram (EEG) was recorded from the scalp, and functional connectivity was computed during the task and used as a predictor of emotional response during the other experimental session. In an immersive simulation session, participants performed a shoot-don’t-shoot scenario while heart rate electrocardiography (ECG) was recorded. Our analysis examined the relationship between the sessions, including behavioral responses (emotional intensity ratings, task performance, and self-report anxiety) and physiology from different modalities [brain connectivity and heart rate variability (HRV)]. Results replicated previous research and found that behavioral performance was modulated within-session based on varying levels of emotional intensity in the laboratory session (*t*_(24)_ = 4.062, *p* < 0.0005) and stress level in the simulation session (*Z* = 2.45, corrected *p*-value = 0.0142). Both behavior and physiology demonstrated cross-session relationships. Behaviorally, higher intensity ratings in the laboratory was related to higher self-report anxiety in the immersive simulation during low-stress (*r* = 0.465, *N* = 25, *p* = 0.019) and high-stress (*r* = 0.400, *N* = 25, *p* = 0.047) conditions. Physiologically, brain connectivity in the theta band during the laboratory session significantly predicted low-frequency HRV in the simulation session (*p* < 0.05); furthermore, a frontoparietal connection accounted for emotional intensity ratings during the attend laboratory condition (*r* = 0.486, *p* = 0.011) and self-report anxiety after the high-stress simulation condition (*r* = 0.389, *p* = 0.035). Interestingly, the predictive power of the brain activity occurred only for the conditions where participants had higher levels of emotional reactivity, stress, or anxiety. Taken together, our findings describe an integrated behavioral and physiological characterization of emotional reactivity.

## Introduction

Emotion is a pervasive component of how we perceive, interpret, and react to the events and interactions of our daily lives, and its pervasive nature likely reflects concomitant responses across physiological systems in brain and body (Lang, 2014). Successful emotion regulation can improve our cognitive and behavioral performance (Lupien et al., 2007) as well as mitigate how we are mentally and physically impacted by stress (Gross and Muñoz, 1995; Gross, 1998; Davidson, 2000). Research has identified several emotion regulation strategies that reduce the intensity of negative experiences (Gross and Levenson, 1993; Ochsner et al., 2004; Etkin et al., 2015), including emotional reappraisal where individuals explicitly reinterpret a negative stimulus or event to be more positive (Richards and Gross, 2000; Gross, 2002; Hajcak and Nieuwenhuis, 2006). Similar strategies to reframe how stressful events are perceived, assessed, and interpreted have also been shown to improve quality of life (Côté et al., 2010), decrease emotional reactivity (Gross and Levenson, 1993; Ochsner et al., 2004; Etkin et al., 2015), and reduce anxiety (Goldin and Gross, 2010).

While the study of emotion encapsulates a rich diversity in psychological constructs, the literature demonstrates strong interrelationships among them, including the three of theoretical interest in our study: emotional reactivity, anxiety, and stress. In research that examined coping strategies, cognitive emotion regulation strategies were related to negative emotional responses including anxiety and stress (Martin and Dahlen, 2005). Similarly, research has shown that negative emotional reactivity is associated with higher anxiety (Boyes et al., 2017) and stress scores (Ripper et al., 2018). Interestingly, these three interrelated, emotional constructs are often associated with a negative valence (Boyes et al., 2017), yet they serve an essential role in adaptation to our environment (Wang and Saudino, 2011; Thayer et al., 2012). For instance, our stress response promotes fast, automatic behavior that does not require cognitive control (Arnsten and Goldman-Rakic, 1998). A stressful event can rapidly increase our heart rate, dilate our pupils, elevate our skin temperature, induce perspiration, and heighten our memory for the experience (Kemeny, 2003). The interrelation among these physiological responses ensures rapid coordination for perception and action (Tsigos et al., 2016), and this is essential to react to unexpected and dynamic events (e.g., recoiling after touching a hot stove, avoiding a collision, or responding to gunfire).

Emotional reactivity can be characterized by peripheral physiological responses (Kemeny, 2003) that are mediated by the autonomic nervous system (ANS; Levenson, 2014). For example, fear is generally accompanied by a withdrawal of parasympathetic activity and an increase in sympathetic activity, which in turn leads to a racing heart, increased sweating, and other peripheral responses that resemble “fight-or-flight.” Research that has linked emotion and ANS has focused on heart rate variability (HRV) as a marker of ANS influence on cardiac activity (Appelhans and Luecken, 2006). When analyzed with spectral analysis, HRV can be decomposed into a low-frequency (LF) component, reflecting a mixture of sympathetic and parasympathetic influences, and a high-frequency (HF) component which reflects purely parasympathetic influences *via* the vagus nerve (Saul, 1990; Reyes del Paso et al., 2013). Importantly, within-person changes in LF and HF-HRV index the autonomic aspects of emotional reactivity (Kreibig, 2010), and HRV captures a relationship between stress response and perception (Barnes et al., 2007), emotional reactivity and/or regulation (Fox, 1989; Appelhans and Luecken, 2006; Mather and Thayer, 2018), and emotional memory (Thayer et al., 2012). Importantly for our research, HRV is a suitable index of ANS responses in contexts with realistic movements and task complexity because it is a non-invasive measure and well established for ambulatory recordings (Laborde et al., 2017).

Additionally, the ANS provides a pathway for bidirectional communication between the viscera and brain. Through this pathway, the ANS and brain work together to coordinate behavioral, experiential, and physiological responses to motivational stimuli—responses that together comprise an emotional reaction (Craig, 2002; Thayer and Lane, 2009; Lang, 2014). Thus, examining HRV alongside brain activity can better reveal the interplay between the central and ANS that give rise to individual differences in emotional reactivity and emotion regulation. HRV is specifically regulated by a *central autonomic* *network* that encompasses both subcortical brain regions (e.g., amygdala, hypothalamus) linked to emotional arousal and basic homeostatic functions as well as higher-order cortical regions (e.g., anterior cingulate cortex) important for emotional appraisal and perception (Benarroch, 1993; Thayer and Lane, 2009). Indeed, HRV has been systematically related to central autonomic brain activity during various emotion inductions (Thayer et al., 2012; Chang et al., 2013; Sakaki et al., 2016), and this bidirectional relationship between ANS and the brain complements neuroimaging work that has identified a core set of brain regions involved with emotion regulation, primarily within the frontoparietal network (Buhle et al., 2014; Kohn et al., 2014). More specifically, connectivity from the frontal cortex has been associated with outcomes related to all three constructs of theoretical interest in our study: emotional reactivity (Domes et al., 2010), anxiety (Kim et al., 2011) and stress (Wang and Saudino, 2011). However, there has been limited research that has examined whether the relationship between HRV and brain responses can capture individual differences in emotional reactivity across different sessions and contexts.

In our study, we examined the relationship between HRV and brain activity, investigating their interdependance as a marker of emotional reactivity between a laboratory setting and an immersive, simulation environment. Each participant completed two experimental sessions. In the laboratory session, participants viewed emotionally-charged images while electroencephalogram (EEG) was recorded from the scalp. We predicted that the neural activity during the task would engage frontal and parietal regions (Domes et al., 2010; Kohn et al., 2014), manifesting in connectivity changes between anterior and posterior electrodes (Aftanas et al., 2002; Zheng et al., 2017). In the simulation session, participants completed an immersive, shoot-don’t-shoot simulation while electrocardiography (ECG) was recorded. Following our previous research (Gamble et al., 2018), we expected that individual differences in HRV would account for variability in performance accuracy during the shoot-don’t-shoot task.

In addition to these predictions for within-session physiological responses, our core analysis examined the relationship between the sessions, including behavioral responses (emotional intensity ratings, task performance, and self-report anxiety) and physiology from different modalities (brain connectivity and HRV). For the behavioral responses, we expected that emotional reactivity would be heightened during the immersive experience (Parsons, 2015); however, the literature is mixed regarding how well behavior in stationary, laboratory tasks predicts task performance when the stimuli have features more typical of real-world environments (Smilek et al., 2008; Hasson et al., 2010; Gramann et al., 2011; Schmälzle et al., 2017; Doré et al., 2018; Wasylyshyn et al., 2018). For the physiological measurements, some recent work has suggested that a stress response can rapidly couple brain and heart responses (Sakaki et al., 2016), but it was unclear whether this relationship would be observed between laboratory and simulation environments, especially with a temporal delay across days. Collectively, our results reveal a robust relationship across tasks and sessions, but only when participants experience higher levels of emotional reactivity as indexed by their behavioral performance and self-report anxiety. Our findings describe an integrated behavioral and physiological characterization of emotional reactivity.

## Materials and Methods

### Participants

This study was carried out in accordance with the accredited Institutional Review Board at US Army Research Laboratory and conducted in compliance with the US Army Research Laboratory Human Research Protection Program (32 Code of Federal Regulations 219 and Department of Defense Instruction 3216.01), with written informed consent from all subjects. All subjects gave written informed consent in accordance with the Declaration of Helsinki. The protocol was approved by the US Army Research Laboratory Human Research Protection Program. We recruited a total of 33 volunteers (32 male, 1 female) from active duty US Army Infantrymen and Special Reaction Teams (SRTs) to ensure that all participants met the minimum requirements to qualify as a marksman. This specific population was necessary because expertise has been shown to influence physiological responses (Johnson et al., 2014), so we wanted to ensure homogeneity across participants in their training for rapid response to dissipate high-threats to better equate skill and physiological response in the shoot-don’t-shoot scenario. We also intentionally used scenes related to military deployments in the laboratory session that were specifically designed to elicit emotional responses from military personnel. Participants with any known heart condition or pace makers were excluded from participating in this study due to the small shock administered as feedback during the simulation session. After providing informed consent, participants completed two sessions (laboratory and simulation).

Data was excluded from participants with excessive signal artifact in the physiological data, or equipment malfunctions during data collection. Additionally, analysis was restricted to male participants to avoid variability due to suggested sex differences in response to negative visual materials (Cahill et al., 2001; Canli et al., 2002), resulting in 25 male participants for analysis (*M* age = 31 years, *SD* = 7.65).

### Laboratory Session: Procedure, Design, and Behavioral Analysis

During the laboratory session, participants sat comfortably at a desk with a computer monitor and keyboard in a sound attenuated chamber. Head measurements from nasion to ion and left preauricular to right preauricular point were taken to size and place a 64-channel BioSemi (Amsterdam, Netherlands) EEG cap, and cleanser was used to prepare the skin around the eyes for VEOG and HEOG external electrodes and behind the ears for reference electrodes on the mastoid bones. BioSemi Actiview software (Amsterdam, Netherlands) was used to ensure electrode impedances were less than 25 kΩ. Participants completed three tasks during the session, but only data from the final task were analyzed here.

Before the task began, the experimenter explained a cognitive reappraisal strategy involving reinterpretation (similar to Ochsner et al., 2004). The experimenter individually presented three demonstration images of aversive events that were not included in the experimental stimulus set, and she guided participants through several examples of how the image could be reappraised so that it no longer elicited a negative response (e.g., reimagining the scene as from a movie, an image from a training exercise to treat wounded Soldiers, or an example of rehabilitative surgery). Following the examples, participants completed three practice trials and verbally described their reinterpretation strategy, allowing for feedback and shaping *via* the experimenter. These practice trials occurred in the same design as the experimental trials so that participants also learned the flow of the trial structure. Once the participant was comfortable with the task, the experimenter left the chamber and started the task.

Using PsychoPy (Pierce, 2007), the negative, emotionally-aversive stimuli from the military affective picture system (MAPS; Goodman et al., 2016) were presented. The MAPS depicts scenes related to recent military deployments, providing a military-experience equivalent to the commonly used International Affective Pictures Set (IAPS; Lang et al., 1997) developed for civilian populations. In the laboratory session, our participants saw each of the 60 high-arousal negative MAPS images in two experimental conditions, Attend or Reinterpret, mirroring an experimental paradigm designed for the IAPS (Hajcak and Nieuwenhuis, 2006).

**Figure 1:**
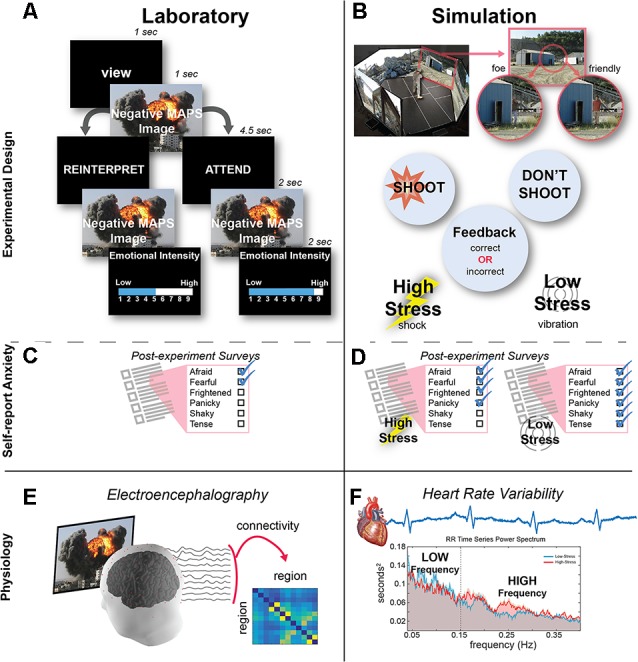
Experimental design. **(A)** In the laboratory session, participants performed an emotion regulation task. After naturally viewing the image during the first presentation, either a REINTERPRET or ATTEND cue appeared to indicate whether the participant should regulate their emotion or naturally view the image during its second presentation. The trial ended after participants rated the emotional intensity during the second image presentation. **(B)** In the 300 degree simulation, participants had to discriminate whether two human characters were both friendlies or one foe and one friendly (depicted). Participants received feedback when they made an error (failing to shoot a foe or incorrectly shooting a friendly): in the high-stress condition, feedback was provided as an electrical shock, while in the low-stress condition, feedback was a vibration. **(C)** At the end of the laboratory session, participants completed a self-report anxiety questionnaire. **(D)** After each simulation condition, participants completed a self-report anxiety questionnaire. **(E)** Brain activity was recorded from the scalp during the laboratory task, and then timeseries data from 12 electrodes were used to compute functional connectivity among each electrode pair. **(F)** Heart activity was recorded during the simulation task, and then heart rate variability (HRV) was computed and averaged to derive the low-fequency (LF) and high-frequency (HF) components.

As illustrated in Figure 1A, each trial began with a 1 s VIEW cue to remind the participant to passively view the image and experience their natural response to the image. After viewing the image for 1 s, a second cue appeared that was either ATTEND or REINTERPRET and remained on the screen for 4.5 s. For Attend trials, participants were asked to just view the image a second time, not altering their natural response to the image. For Reinterpret trials, participants were asked to reappraise the image to change their emotional response so that the image no longer elicited a negative response. The second presentation of the image lasted for 2 s. To end the trial, participants used the numeric keypad on the keyboard to rate their emotional intensity in response to each image on a Likert-scale ranging from 1 (low emotional intensity) to 9 (high emotional intensity). The inter-stimulus interval between trials was 0.5 s, and the participants were given two breaks (every 20 trials) and resumed when ready by pressing the space bar. Unlike the practice trials, participants did not give any verbal responses during the experimental portion of the task. For the analysis, the average emotional intensity score was computed separately for the Attend trials and the Reinterpret trials and used to index the emotional reactivity experienced in each experimental condition (Figure 1A).

After rating all 60 images, participants completed the Multiple Affect Adjective Checklist-Revised (MAACL-R) Today form (Lubin and Zuckerman, 1999). The MAACL-R Today form is a state measure consisting of 132 adjectives, and this self-report questionnaire has been used to capture affective responses to military-relevant stressful situations (Fatkin et al., 1991; Hudgens et al., 1991; Patton and Gamble, 2016). Participants were asked to mark all of the words that described how they “feel right now” to capture a self-report of their emotion after viewing all of the negative images. Our analysis focused on the MAACL-R Anxiety subscale (Figure 1C), consisting of 10 adjectives (e.g., afraid, fearful, frightened, panicky, shaky, tense). Raw scores were calculated by counting the number of adjectives endorsed from this subscale and then transformed based on the total number checked to standard T-scores provided by the survey developers and normed for male participants.

### Laboratory Session: Brain Analysis

Continuous EEG recordings were captured *via* the Biosemi ActiveTwo Bioamplifier system (Amsterdam, Netherlands) equipped with standard Ag/AgCI electrodes from 64 sites on the scalp. Raw EEG measurements were pre-processed using in-house software in MATLAB (Mathworks, Inc., Natick, MA, USA) and the EEGLAB toolbox (Delorme and Makeig, 2004). The pre-processing pipeline largely follows the PREP approach (Bigdely-Shamlo et al., 2015) and contains five steps: (1) resampling the raw EEG to 250 Hz; (2) line noise removal *via* a frequency-domain (multi-taper) regression technique to remove 60 Hz and harmonics present in the signal; (3) a robust average reference with a Huber mean; (4) artifact subspace reconstruction to remove residual artifact (the standard deviation cutoff parameter was set to 15); and (5) a piece-wise detrending algorithm to remove LF drift in the signal (window size = 330 ms, step size = 8 ms).

After preprocessing, we used the Source Information Flow Toolbox (SIFT; Mullen, 2014) in EEGLAB to estimate the direct Directed Transfer Function (dDTF) using the “Ridge Regression” algorithmic option for fitting the vector autoregressive model (Order = 15) across the full-length of the timeseries during the MAPS task. The dDTF method of directed connectivity allows for short time windows of EEG connectivity whilst also overcoming (in part) the ambiguity between direct and indirect influences of connections (Korzeniewska et al., 2003). For computational efficiency and to mitigate against over-fitting, we reduced the model from 64 to 12 channels spanning the entire brain (CP3, CPz, CP4, P3, Pz, P4, FC5, F7, F3, FC4, F8, F4).

Connectivity was computed between all pairs, yielding 132 total connections from each of the 12 channels to each of the other 11 channels (Figure 1E). The dDTF estimates were then averaged within the four common frequency bands: delta: 1–3 Hz, theta: 4–7 Hz, alpha: 8–12 Hz, and beta: 13–25 Hz. We then used these values in two analyses:

1. To inspect the differences in dDTF estimates across frequency bands and between conditions (Figure 3), we baseline corrected the dDTF estimates by subtracting the mean dDTF 250 ms before the onset of the second image (during the second cue, see Figure 1A), and then we averaged the standardized (z-score) dDTF estimates across subjects.
2. For the LASSO regression analysis, the dDTF estimates were averaged over the 2-s duration of the second presentation of the image, capturing the brain activity specific to the attend or reinterpret viewing of the negative MAPS image (Figure 1A).

**Figure 2:**
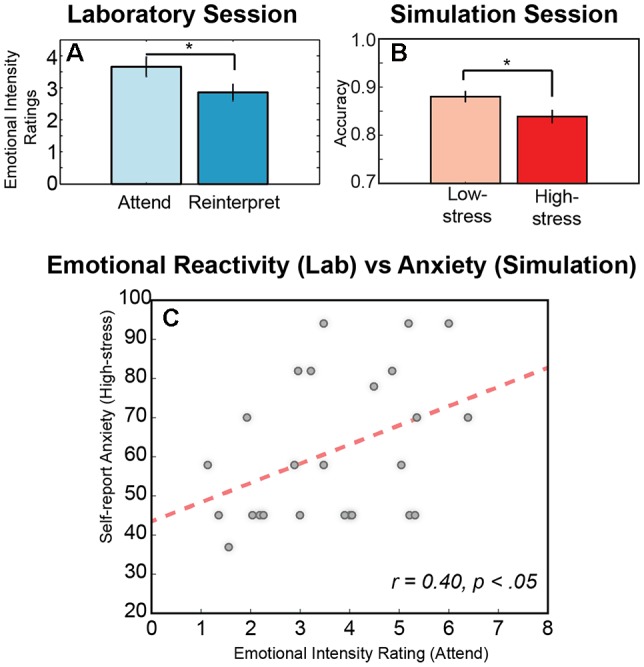
Behavioral responses across sessions. **(A)** Significant difference in the mean intensity ratings for the negative military affective picture system (MAPS) images averaged across conditions (attend, reinterpret) and across participants. **(B)** Significant difference in the mean accuracy for the immersive shooting task averaged across conditions (low-stress, high-stress) and across participants. **(C)** Significant relationship between emotional intensity rating during the attend condition of the laboratory emotion regulation task and the self-report anxiety score after the high-stress in the simulation environment. An * indicates significance in a pair wise *t*-test (*p* < 0.05), and error is indicated as SEM across participants.

**Figure 3:**
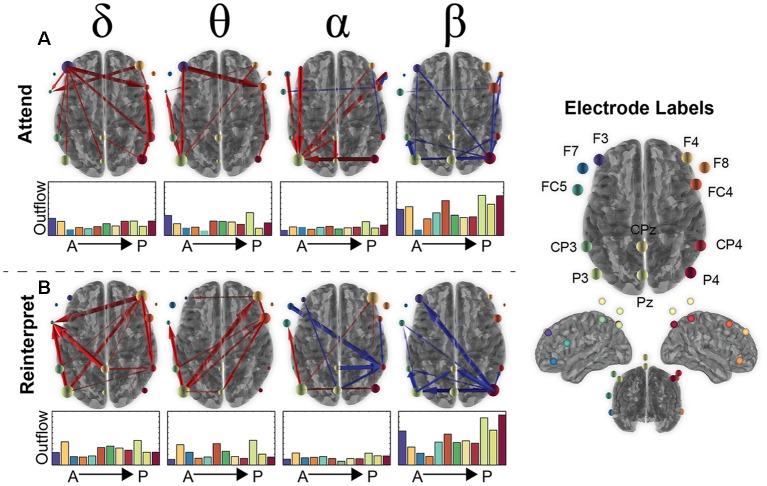
Brain connectivity changes during the second image presentation in the emotion regulation task. **(A)** The top 90% of connectivity changes in the Attend condition for the 12 electrodes (labels shown on the right) for each frequency band of interest (delta, theta, alpha, beta). Red arrows signify an increase in connectivity compared to baseline (250 ms before onset of second presentation) and blue a decrease from baseline. The total “outflow” (or summed connectivity) for each electrode is visualized by scaling the orb size as well as bar plots under each axial brain image that are organized from anterior to posterior electrodes (color of bar plot matches the color of the circle in the electrode label picture). **(B)** The same information for the Reinterpret condition as shown in **(A)** for Attend.

In sum, each trial was represented as four sets of 132 connections, one for each frequency band, and we then separately averaged over trials for each condition, attend and reinterpret. This resulted in a 132 (connections) × 4 (frequency) × 2 (condition) matrix for each participant.

### Immersive Simulation Session: Procedure, Design, and Behavioral Analysis

In the simulation session, participants moved freely and untethered around a 300-degree immersive, virtual environment that was implemented using the VirTra V 300™^1^ system and projected on five 6′ × 10′ screens (Figure 1B). Building on previous research (Patton, 2014), the shoot-don’t-shoot scenario was presented in a panoramic view of a quarry environment, and an auditory tone indicated when and where two human characters would appear simultaneously from behind objects in the quarry. The characters were either: (1) two friendly actors, both holding innocuous objects (e.g., cell phone or soda can) or with their hands up; or (2) a mix of one friendly and one foe shooting at the participant with a black pistol in their hand. Participants had to rapidly discriminate friendly or foe in the 2 s that the actors appeared on the screen. To resolve each presentation, participants were instructed to withhold fire when the character was a friendly and fire when the character was a foe. To disarm the foes, participants used an untethered modified M-4 carbine rifle, the standard issue in the U.S. Army. The rifle was modified to provide realistic weapon feedback from recoil generated from carbon dioxide release from the ammunition magazines, safely providing feedback similar to an unmodified weapon, and it was outfitted with a laser to track the location of fire in the virtual environment. Participants also felt feedback on their waist from a VirTra Threatfire™ device placed on their waistline at belt level when: (1) a foe in the environment successfully shot them (miss); or (2) they incorrectly shot a friendly (false alarm). They received no feedback when they successfully disarmed a foe (hit) or withheld fire from a friendly (correct reject).

After a training session to become familiar with the environment, participants completed 256 trials, where half of the trials consisted of two friendly actors appearing and the other half consisted of one friendly and one foe. These trials were then equally split across two experimental conditions (128 trials each), a low-stress vibration condition and a high-stress shock condition (Figure 1B). In the low-stress condition, participants received a 500 ms vibration, similar to that of a cell phone, from the VirTra Threatfire™ device when they made an error. In the high-stress condition, feedback was given through the VirTra Threatfire™ shock belt, which delivered a 200 ms, 50 mA electric shock, administered on alternating sides of the waist when they made an error. The order of conditions was counterbalanced between participants, so while a learning effect was observed between first and second condition (accuracy: *t*_(25)_ = −5.51, *p* < 0.001), performance was otherwise equivalent within the low- and high-stress trials. For safety, the feedback in both conditions was configured to ensure that at least 60 s occurred between vibration (low-stress) or shock (high-stress) feedback events. During this 60 s, both friendly and foe presentations could occur, but if contact was made by the participant, no feedback was administered other than seeing targets disappear or fall down.

The accuracy on each trial was determined by the first shot fired and mirrored the feedback received: hit for shooting at a foe (H), miss for not shooting at a foe (M), false alarm for shooting at a friendly (FA), and correct rejects for not shooting at a friendly (CR). After computing z-scores for each category (Stanislaw and Todorov, 1999), accuracy was defined as Hits plus Correct Rejections divided by the number of trials (*N* = 128) and computed separately for each condition (low-stress and high-stress):

$$\left(1\right)accuracy=\frac{\left(H\right)+(CR)}{N}$$

Similar to the laboratory session, participants completed the MAACL-R Today form (Lubin and Zuckerman, 1999) in the simulation session, once after the low-stress condition and once after the high-stress condition (Figure 1D). Participants were asked to mark all of the words that described how they “felt during the simulation” to capture a self-report of their emotion during each of the stress conditions. Again, our analysis focused on the MAACL-R Anxiety subscale, and the score was computed using the same method as described in the laboratory section.

### Immersive Simulation Session: Heart Rate Analysis

Continuous ECG recordings were captured *via* the Equivital™ EQO2 and LifeMonitor belt. Raw ECG measures were pre-processed using automated algorithms in vivonoetics’ monitoring and analysis software, Vivosense^®^, using the parameters of heart rate above 200 or below 40 beats per minute, high noise filtering, and removing ectopic beats.

After preprocessing, the R–wave timeseries estimated HRV as the temporal differences between heart beats over time, and this timeseries was interpolated and resampled to 4 Hz, and a fast fourier transform (FFT) was performed on the time series data between 0.04 and 0.4 Hz (Figure 1F). We then averaged with the two common frequency bands: LF: 0.04–0.15 Hz and HF: 0.15–0.4 Hz bands. The HRV values were computed separately for the low-stress and high-stress experimental conditions, creating a 2 (frequency range) × 2 (condition) matrix for each participant.

### Cross-Session Analysis: Brain Response Predicting Heart Rate Variability Response

Following previous research (Mwangi et al., 2014; Powell et al., 2018), we employed a LASSO regression framework (Tibshirani, 1996, 2011) to examine the predictive relationship between the brain connectivity in the laboratory session and the HRV responses in the simulation session. The 132 directed neural connections were used as predictors of the HRV response, and LASSO identified the most predictive connections and zeroed out the irrelevant ones by penalizing regression coefficients based on the regularization constant lambda.

We computed separate models for each combination of laboratory condition (attend, reinterpret), EEG frequency (delta, theta, alpha, beta), simulation condition (low-stress, high-stress), and HRV frequency (LF and HF). We used a Monte-Carlo cross-validation to assess the robustness of each model fit. For each combination of brain and heart data, we ran 500 Monte-Carlo iterations where participants were randomly split in a training set (20 participants) and test set (five participants), and the model fit to the training set was used to predict the brain-heart relationship of the test set and assess the robustness of the relationship. Since the regularization constant lambda was also a parameter to fit, we tested each of the brain-heart rate models at each of 25 regularization constants in logarithmic steps from 0.032 to 1. The score for each of the 25 lambda values was the mean out-of-sample *R*^2^ score across its 500 splits.

Thus, the cross-validated LASSO regression models identified what neural connections in the laboratory were predictive of the HRV in the simulation environment, how robust this relationship was across participants and lambda parameter values, and provided a percent variance of HRV that was accounted for by each significant brain connection.

### Cross-Modal Analysis: Linking Brain to Behavior

From the LASSO analysis, results identified 16 connections that were robust across the model fits and significantly related to LF-HRV. In our final analysis, we examined the relationship of these significant brain connections and our behavioral performance and self-report anxiety metrics. We separately assessed the correlation between the brain data and the following: lab emotional intensity attend, lab emotional intensity reinterpret, lab self-report anxiety, simulation accuracy low-stress, simulation accuracy high-stress, simulation self-report anxiety low-stress, and simulation self-report anxiety high-stress. Correlational analyses were completed between these 16 connections and the behavioral responses in the statistical package for the social sciences (SPSS).

## Results

### Behavioral Responses of Emotional Reactivity Observed in Laboratory and Simulation

We first examined the behavioral responses in the two sessions to assess whether the experimental conditions modulated task performance. In the laboratory task, participants viewed each negative MAPS image in one of two experimental conditions, Attend or Reinterpret (Figure 1A). In the Attend condition, participants were asked to attend naturally to the stimulus, reporting the overall emotional intensity that they experienced when viewing the image (*M* = 3.65, *SD* = 1.52). In the Reinterpret condition, participants were asked to reinterpret the image during a short delay period and then report the emotional intensity they experienced after regulating their emotional response to the image (*M* = 2.85, *SD* = 1.27). As illustrated in Figure 2A, the average intensity for the Attend condition was higher than for the Reinterpret condition, and this difference was statistically significant (*p*_(24)_ = 4.062, *p* < 0.0005). This result indicates that emotional reactivity was successfully modulated during the two experimental conditions in the laboratory session.

Similarly, for the simulation session, we compared performance accuracy in the shoot-don’t-shoot task between the low-stress and high-stress conditions. In both conditions, participants received feedback for errors when they failed to shoot a foe or incorrectly shot a friendly; however, in the low-stress condition, the participants felt a vibration, while in the high-stress condition, they received a shock. As illustrated in Figure 2B, performance accuracy decreased in the high-stress condition (*M* = 0.84, *SD* = 0.06) compared to the low-stress condition (*M* = 0.87, *SD* = 0.05), and as expected, this difference was statistically significant (*Z* = 2.45, corrected *p*-value = 0.0142). Similar to the laboratory session, this result demonstrates an effect of experimental condition in the expected direction, where high-stress negatively impacts performance.

Next, we investigated the anxiety score derived from the MAACL-R and examined its variability between sessions. Overall, participants reported statistically significant lower anxiety during the laboratory session (*M* = 47.88, *SD* = 8.671) compared to the simulation session, both for the low-stress simulation condition (*M* = 58.20, *SD* = 19.59; *t*_(24)_ = 2.835, *p* = 0.009) and the high-stress simulation condition (*M* = 61.40, *SD* = 18.59; *t*_(24)_ = 3.404, *p* = 0.002). This stable difference in self-report anxiety between sessions likely reflects the value of the immersive and more realistic experience provided in the simulation session to induce emotional processes of interest (Parsons, 2015). However, results also demonstrated that the emotional intensity ratings provided in the laboratory session accounted for variability in the self-report anxiety in the simulation session (Figure 2C). Specifically, participants who reported a higher level of perceived emotional intensity in response to naturally attending to the negative images viewed in the laboratory session also reported higher levels of anxiety in both the low-stress (*r* = 0.465, *N* = 25, *p* = 0.019) and high-stress conditions (*r* = 0.400, *N* = 25, *p* = 0.047) of the simulation session.

Overall, these results confirm that our experimental conditions modulated performance metrics that index emotional reactivity, namely the emotional intensity ratings in the attend/reinterpret conditions in the laboratory, and task accuracy in the low-stress/high-stress conditions in the immersive simulation. Results also demonstrated a relationship between sessions, revealing that higher emotional intensity reported during the attend condition of the laboratory task related to higher self-report anxiety in the virtual shoot-don’t-shoot scenario. Together, these behavioral results suggest an interdependance among our three constructs of interest: emotional reactivity, stress, and anxiety. We next examine the neuroimaging data to investigate variability in the underlying physiological response to these constructs.

### Brain Activity Observed in Regions Associated With Emotion Regulation

Using the neuroimaging data recorded during the laboratory task, we analyzed the neural activity during the response to the second presentation of each negative MAPS image to estimate neural processing related to the emotional response on each trial. We computed pairwise functional connectivity among 12 channels to estimate the causal influence between the neural activity recorded by each electrode (132 connections total). Connectivity relationships were computed separately for each condition (attend, reinterpret) and for four frequency bands (delta, theta, alpha, and beta).

Based on the magnitude or strength of the pairwise relationship, the top 10% of connections were plotted in Figure 3, with red arrows signifying an increase in connectivity and blue a decrease compared to baseline. Although there was some variability in what electrodes are involved between conditions, the frequency bands showed a similar pattern with overall increased connectivity in the slower frequency bands (delta, theta) and decreased activity in the fastest frequency band (beta). Alpha connectivity showed a difference, with increased activity in the attend condition and more decreased activity in the reinterpret condition.

In Figure 3, the connectivity patterns were also summarized in terms of their overall “outflow” defined as the summed connectivity magnitude of all connections emanating from a given electrode. This metric can be interpreted as the amount of influence that a given electrode has on all other electrodes. The amount of outflow for each electrode was visualized in Figure 3 as a change in size of the orb for each electrode as well as bar plots shown underneath each brain image.

Across conditions and frequencies, four electrodes routinely arose as the ones with the highest outflow. In the beta band, P4 had the largest decrease in outflow across both conditions (Attend: −0.37; Reinterpret: −0.47), suggesting a suppression of activity that is slightly greater during the reinterpret condition. This might indicate the beta band from parietal cortex has a role to suppress the emotional response when asked to reinterpret negative images. For the other three frequency bands, the P3 electrode had the highest outflow (theta: 0.22, delta: 0.18, alpha: 0.11), with the F3 and F4 electrodes a close second (left F3 in attend condition—theta: 0.16, delta: 0.19, alpha: 0.05; and right F4 in reinterpret condition—theta: 0.22, delta: 0.19, alpha: 0.11). This effect was strongest in the theta band in the attend condition, suggesting that the frontal cortex may strongly influence the emotional intensity experienced during the passive viewing of a negative image.

Overall, both conditions recruit regions that span parietal and frontal areas as expected from previous emotion regulation literature (Buhle et al., 2014; Kohn et al., 2014), and there were not statistically significant differences in the connectivity between attend and reinterpret conditions (FDR > 0.05). In contrast, frequency bands demonstrate variability in both the regions involved as well as whether there is increased or decreased connectivity compared to baseline. We next examined whether these brain connectivity differences in the laboratory session can predict HRV response differences in the simulation session.

### Laboratory Physiology Predicts Simulation Physiology

**Figure 4:**
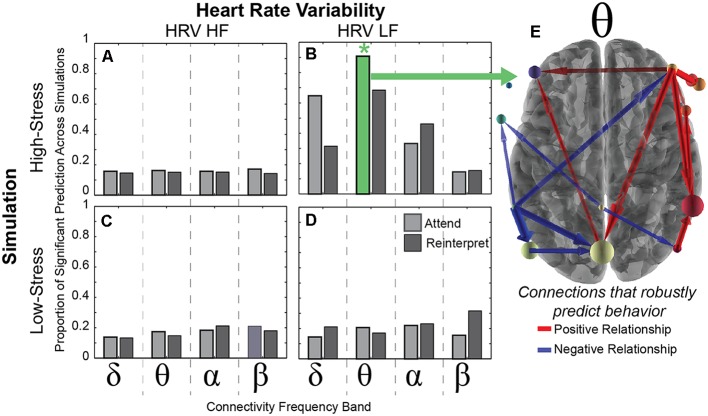
Brain connectivity predicts HRV. **(A–D)** Each bar represents the proportion of positive *R*^2^ random samplings found with the cross-validation procedure (see “Materials and Methods” section) within each frequency band (delta, theta, alpha, beta) that predicts HRV (HF on left and LF on right) in both simulation conditions (high-stress in top row and low-stress in bottom row). The green *indicates a significant relationship between the theta band connectivity and the LF-HRV in 95% of the random iterations across 500 cross-validation folds. **(E)** The theta connections that significantly predict LF-HRV in the high-stress condition, with positive relationships in red and negative relationships in blue. Each arrow represents the direction of connection, scaled by the number of times that the connection is non-zero across Monte-Carlo simulations.

We utilized a LASSO regression framework to examine the predictive relationship between the 132 brain connections in the laboratory session and the HRV responses in the simulation session. As shown in Figure 4, we computed separate models for each combination of laboratory condition (attend, reinterpret), EEG frequency (delta, theta, alpha, beta), simulation condition (low-stress, high-stress), and HRV frequency (LF and HF), and we evaluated the robustness of the relationship using a Monte-Carlo cross-validation analysis.

Results revealed that the neural connectivity in the theta band while viewing negative images in the attend condition during the laboratory session predicted LF-HRV during the high-stress condition of the immersive threat discrimination task (Figure 4). This relationship was robust to the 500 cross-validation iterations, indicating that the theta band connectivity pattern significantly accounted for variability in the LF-HRV in 95% of the random iterations (marked by a positive *R*^2^ value). Furthermore, this significantly predictive relationship was consistent across models using 76% of regularization constants tested, where variance in connectivity in the theta band during the attend condition explained at least 40% of the variance in LF-HRV during the high-stress condition in the simulation. No other model was significant.

We next examined which of the 132 connections accounted for the LF-HRV response variability. The robustness of the connection was determined by counting the number of iterations (within the 95% showing a positive *R*^2^) for which the connection contributed to the positive *R*^2^, and results identified 16 connections that significantly contributed to the predicted relationship. In Figure 4, wider arrows indicate a greater number of models in which the connections predicted LF-HRV, and color corresponds to the sign of the majority of the regression coefficients, with red indicating a positive and blue a negative value.

In short, these connections show a robust relationship with LF-HRV, implicating both theta band activity and LF-HRV as physiological markers of emotional reactivity when responding to aversive stimuli. Importantly, this predictive relationship is robust to task and context, and the relationship is stable across different days indicating the reliability of these physiological responses.

### Frontoparietal Connection Linked to Behavioral Responses

In our final analysis, we investigated whether the robust relationship in physiology between the laboratory and simulation session can also account for the behavioral responses. Using the 16 connections significantly related to HRV, we use a correlational analysis to relate the significant brain connections to all seven of our behavioral measures: the emotional intensity ratings in the laboratory (attend, reinterpret), the laboratory self-report anxiety, the performance accuracy in the simulation (low-stress, high-stress), and the simulation self-report anxiety (low-stress, high-stress).

**Figure 5:**
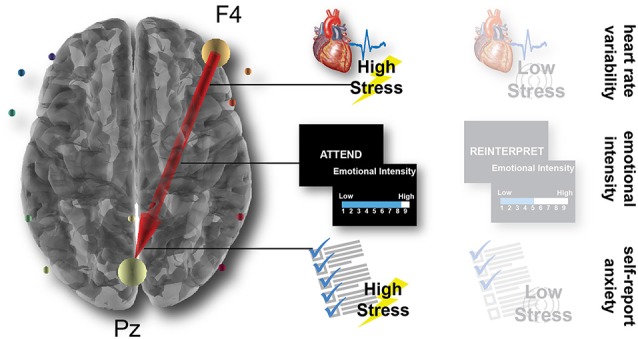
Brain connectivity predicts behavioral and physiological outcomes. Across the LASSO regression and subsequently correlational analyses, a single connection from the anterior F4 electrode to the posterior Pz electrode predicts the LF-HRV in the high-stress simulation condition, the emotional intensity ratings in the attend laboratory condition, and the self-report anxiety in the high-stress simulation condition.

As illustrated in Figure 5, results demonstrated that one connection from a right anterior electrode to a central posterior electrode accounted for variance in two of the behavioral metrics: emotional intensity ratings in the attend condition of the laboratory task (*r* = 0.486, *p* = 0.011) and self-report anxiety after the high-stress condition in the immersive simulation (*r* = 0.389, *p* = 0.035). More specifically, the outflow from F4 (likely related to brain activity in the frontal cortex) to Pz (likely related to activity in parietal cortex) accounts for approximately 24% of the variance in emotional intensity ratings in the attend condition and 15% of the variance in the self-report anxiety in the high-stress condition.

The robust relationship of this F4-Pz connection with multiple measures that likely index emotional reactivity aligns with previous research that has found a critical role for the frontoparietal network in successful emotion regulation (Buhle et al., 2014; Kohn et al., 2014). Importantly, this neural connectivity is linked with our metrics from the experimental conditions with the highest levels of emotional reactivity: the HRV in the high-stress but not low-stress condition, the emotional intensity in the attend but not the reinterpret condition, and the self-report anxiety after the high-stress but not the low-stress condition. This relationship manifests in metrics from within the same session (brain predicts emotional intensity in the laboratory) as well as metrics between sessions, contexts, and days (brain predicts HRV and self-report anxiety in the simulation).

## Discussion

In this study, we examined emotional reactivity and its interrelationships with stress and anxiety across two experimental sessions. Military and SRT participants completed an image viewing task of negative MAPS pictures in the laboratory on 1 day, and then they completed an immersive shoot-don’t-shoot task in a virtual simulation on another day. Each task included two experimental conditions, and our behavioral results confirmed that task performance was modulated across conditions in the expected direction: participants rated negative images with higher emotional intensity in an attend condition compared to a reinterpret condition, and participants were less accurate disarming foe characters in a high-stress condition compared to a low-stress condition. Their self-reported anxiety was also higher in the simulation scenario, suggesting an effect of immersion (Patton, 2014; Parsons, 2015); however, stability in their emotional responses across sessions was also observed. Specifically, higher ratings of emotional intensity in the laboratory were statistically related to higher self-report anxiety in the immersive simulation.

This behavioral relationship between the laboratory and simulation was mirrored in a robust, yet specific, relationship between the brain connectivity in the laboratory and the heart response in the simulation. Interestingly, this relationship was strongest in the theta band during the attend condition, where participants had higher emotional intensity ratings, and the LF-HRV during the high-stress condition, where participants had decreased performance accuracy. This suggests that the relationship may be salient only in the conditions that invoke higher emotional reactivity. Complementing this result, a dependence on higher emotional content was also revealed in the correlation between brain connectivity in the F4-Pz connection and emotional intensity ratings during the attend condition and self-report anxiety after the high-stress condition. Together, our findings describe an integrated behavioral and physiological characterization of emotional reactivity.

### Frontoparietal Connectivity Relates to Emotional Reactivity

Our theoretical interest centered on emotional responses and their physiological interdependances across contexts. More specifically, we studied the relationship between a standard laboratory emotion regulation task and its relationship to the embodied experience of stress in an immersive shoot-don’t-shoot task. Results revealed a strong link between a frontoparietal connection across tasks and contexts, showing the strongest relationship during experimental conditions where participants experienced higher levels of emotional intensity, stress, or anxiety. This finding is well aligned with previous literature that has implicated frontoparietal involvement during emotion regulation (Buhle et al., 2014; Kohn et al., 2014), including a set of studies where connectivity from frontal cortex was associated with outcomes related to emotional reactivity (Domes et al., 2010), anxiety (Kim et al., 2011), and stress (Wang and Saudino, 2011). Interestingly, the EEG network activity was concentrated over the F4 electrode which has been previously associated with activity from the dorsolateral prefrontal cortex (DLPFC; Herwig et al., 2003). This frontal region has been implicated in both emotion reappraisal (Phillips et al., 2008) and risk-taking behavior (Fecteau et al., 2007), and our tasks sit at their intersection as an emotionally-charged, risky decision to shoot or not shoot. The physiological interdependance across contexts suggests that emotion regulation may rely on an implicit assessment of risk, where the risk of adapting an emotional response to an event may be evaluated in relation to its impact on the success in future behavior. Research to explore how risk and emotion regulation may invoke complementary processes in the DLPFC is an exciting avenue of interest for future research.

Our results demonstrated that this predictive relationship was specific to the theta band, an intrinsic oscillation often implicated in a variety of cognitive processes. The theta band has been found to play a critical role in episodic memory formation and general working memory function (Klimesch, 1999; Aftanas et al., 2002), two cognitive processes heavily influenced by emotional responses. Moreover, the theta band has also been linked to anxiety while appraising visual stimuli (Aftanas et al., 2002), corroborating our result that links theta activity to self-report anxiety in the immersive simulation. However, theta activity in our study was also related to the more general experience of high emotional intensity reported after viewing the MAPS images, suggesting that theta activity in the frontoparietal network may reflect the entanglement of emotion in other cognitive processes.

### Robust Prediction of LF-HRV in Simulation From Theta Activity in Laboratory

While neuroimaging research has identified neural correlates of emotional reinterpretation, our work extends our understanding of emotional reactivity by examining the interrelationships between brain and HRV between tasks, contexts, and days. Our results specifically linked neural connectivity in the theta band to LF-HRV. Research in the last decade suggests that the specific autonomic contributions to LF-HRV are ambiguous, suggesting it is dually influenced by some mix of sympathetic and parasympathetic influences (Randall et al., 1991; Reyes del Paso et al., 2013); however, research has found LF-HRV increases in response to negative emotion (Sloan et al., 1994; Murakami and Ohira, 2007), and individuals showing high levels of negative emotionality (e.g., anxiety patients, high trait anger) show greater levels of LF-HRV compared to their low LF-HRV counterparts (Ottaviani et al., 2009; Shah et al., 2013). Our research aligns with this previous research because the predictive brain-heart relationship was observed only in the high-stress condition of the simulation, the condition that participants reported higher levels of negative emotion.

LF-HRV has also been associated with connectivity between neural regions implicated in emotional processing, including amygdala, dorsal anterior cingulate, ventromedial PFC, and the temporoparietal junction (Chang et al., 2013; Thome et al., 2017). A proposal by Chang et al. (2013) posits that connectivity in the emotional network that co-occurs with changes in LF-HRV could in principle be linked to an adaptive process in which psychological and physiological states are adjusted when reorienting attention and changing emotional states. Our results are a novel extension of this interrelationship of brain connectivity and LF-HRV, showing that this predictive relationship is stable between tasks as well as across days. This may reflect that these physiological metrics are capturing the intrinsic, stable processing of emotional reactivity, but future research is needed to further address the generalizability of this relationship across contexts and time.

### Understanding the Link Between the Laboratory and Immersive Simulation Outcomes

Our experimental design explored the relationship between a controlled laboratory session and an immersive simulation session, and our findings describe an integrated behavioral and physiological characterization of emotional reactivity. The emotion regulation paradigm included in our study has been primarily studied within a controlled laboratory setting using emotion invoking stimuli (Jackson et al., 2003; Ochsner et al., 2004; Domes et al., 2010; Thiruchselvam et al., 2011; Uchida et al., 2015). Our work aligns with the proposal that incorporating additional realistic and complex experimental environments may provide a more enhanced model of emotion regulation beyond what has been shown in controlled laboratory studies alone (Parsons, 2015).

Additionally, recent neuroscience research has shown that non-invasive neural measurements made in the laboratory may be used to predict behavioral outcomes in the real world across a variety of tasks and diverse contexts (Tompson et al., 2019). This framework has been coined the brain-as-predictor approach (Berkman and Falk, 2013), and the promise of the approach has been shown in a variety of tasks, including second language acquisition (Tan et al., 2011), intelligence quotients (Choi et al., 2008), and high-risk driving behavior (Wasylyshyn et al., 2018). We employed this approach to compare emotional reactivity during a stationary laboratory task to an immersive simulation scenario. The simulator provided the opportunity for participants to move around in the environment and receive physical feedback, providing a closer approximation to a real-world experience. Our results demonstrated that connectivity strength in a frontoparietal network while viewing negative, high arousal images predicts several indices of a participant’s emotional response in an immersive shoot-don’t-shoot scenario: LF-HRV and self-report anxiety after a high-stress condition. Interestingly, this brain-as-predictor relationship was significant for emotional responses measured on different days in different contexts. More generally, our findings further add to the growing literature that psychological studies broaden the scope beyond the laboratory for extended validity and generalizability to real-world outcomes (Kingstone et al., 2003; Doré et al., 2018; Schmälzle et al., 2017).

### Methodological Considerations

Our results indicate the promise of linking physiological responses across contexts and days; however, a few limitations resulting from design decisions in our work indicate several interesting directions for future research. While the differences between the laboratory and simulation tasks had the benefit of examining generalizability of the core constructs examined, these discrepancies may also limit the direct mapping of constructs between the two scenarios. Though both the MAPS and simulation scenario stimuli were designed to induce stress and negative affect, participants may vary in their response to these stimuli across intensity, type, and duration. That is, individuals may vary in how they respond to viewing emotionally-aversive picture stimuli compared to their emotional response when immersed in a shoot-don’t-shoot task with physical feedback. Furthermore, the physiological measurement of this reactivity had to vary based on methodological considerations. The amount of physical movement during the immersive simulation session prevented the collection of EEG, so our research extends literature that has linked both brain activity and HRV to emotion (Chang et al., 2013; Sakaki et al., 2016). Future research could employ laboratory and immersive scenarios with higher interdependency in stimuli and/or task to examine whether more similarity in scenarios enhance the relationships found between sessions.

Additionally, our analysis was limited to male participants with expert training in reacting to high-stress situations. Previous research has identified sex differences in response to negative visual materials (Cahill et al., 2001; Canli et al., 2002) and in brain activation patterns elicited from emotional stimuli (Wager et al., 2003), all while expertise also shapes our neural responses to stimuli (Gauthier et al., 2000). Thus, based on our population, we may have limited the generalizability of our results, and future work in this area may aid in developing broader characterizations of emotional reactivity and regulation.

Our study did not continually monitor the employment of the reinterpretation strategy during the laboratory task. Participants only verbalized their strategy during training, but not during the experiment. Thus, we do not know if participants continued to employ this strategy during the MAPS image task. Future studies using the brain-as-predictor approach may find that regulation strategy plays a key role to isolate the neural networks serving this emotion regulation task and may better predict behavioral and physiological outcomes across contexts.

Finally, due to model constraints, we only inspected a subset of the connectivity patterns available and restricted our analysis to a priori bands of interest, despite the inherent complexity of brain signals (Buzsáki, 2006). Thus, future studies may show that the connectivity pattern shown to reliably relate to a variety of measurements may actually only be a subset of connections within an intricate mesh of brain connections that may predict the behavioral outcomes explored.

## Author Contributions

HR, KG, DP and JV contributed to the design of the study and/or collected data. HR, NW, DS, KG, JB, JG and JV contributed to the analytic approaches, interpreted the findings and substantially edited the article. HR, NW, DS and JG analyzed data. HR, NW, JG and JV wrote the article. All provided feedback on the article.

## Conflict of Interest Statement

The authors declare that the research was conducted in the absence of any commercial or financial relationships that could be construed as a potential conflict of interest.

## Footnotes

^1^http://www.virtra.com/v-300
